# Supplementary material for: Post-transcriptional regulation of cyclin A and cyclin B mRNAs is mediated by Bruno 1 and Cup, and further fine-tuned within P-bodies
Source: bioRxiv. 2024 Oct 17:2024.10.17.618951. Preprint. [Version 1] doi: 10.1101/2024.10.17.618951 (PMC11507948; doi:10.1101/2024.10.17.618951)
Supplement: Supplementary file 1 [file media-3.docx]

**Supplemental Figure Legends**

**Figure S1. Bru1 and Cup knockdowns lead to re-entry of nurse cells into the cell cycle and to the ectopic expression of CycA and CycB proteins.** 

**(A)** ɑ-tubulin visualized via IF in stage 6 egg chambers expressing Cup-YFP and Bru1-GFP in the indicated backgrounds. Membrane (WGA). Images are deconvolved, XY max-intensity Z-projections of 5 optical slices (0.3µm each). Scale bars, 10μm.

**(B)** ɑ-tubulin visualized via IF in a chain of egg chambers expressing Cup-YFP and Bru1-GFP in *cup*^*RNAi*^ background. Membrane (WGA). Red arrows indicate the re-formation of the nuclear membrane. Images are deconvolved, XY max-intensity Z-projections of 6 optical slices (0.3µm each). Scale bars, 50μm.

**(C)** Immunoblot against CycA and CycB. H3 - the loading control using lysates prepared from egg chambers in the indicated backgrounds. 

**Figure S2. Bru1 and Cup form large complexes with** ***cycA*** **and** ***cycB*** **mRNAs in the nurse cell cytoplasm.**

**(A)** Visualization of *cycA* and *cycB* mRNAs in a chain of egg chambers. F-actin stain to outline the egg chambers. Images are deconvolved, XY max-intensity Z-projections of 10 optical slices (1µm each). Scale bar 50μm.

**(B)** Co-visualization via STED of *cycA* and *cycB* mRNAs in the nurse cells of a sectioned egg chamber at mid-oogenesis expressing Bru1-GFP and Cup-YFP. White box indicates the location of the zoomed in images in Figure 2A. Images are deconvolved, XY max-intensity Z-projections of 3 optical slices (0.1µm each). Scale bar 5μm.

**(C)** Co-visualization via STED of *cycA* and *cycB* mRNAs in the nurse cells of a sectioned egg chamber at mid-oogenesis expressing Bru1-GFP. Colored dots indicate the individual channels merged per image. White box indicates the location of the zoomed in images. Images are deconvolved, XY max-intensity Z-projections of 3 optical slices (0.1µm each). Scale bar 5μm.

**(D)** Co-visualization via STED of *cycA* and *cycB* mRNAs in the nurse cells of a sectioned egg chamber at mid-oogenesis expressing Cup-YFP. Colored dots indicate the individual channels merged per image. White box indicates the location of the zoomed in images. Images are deconvolved, XY max-intensity Z-projections of 3 optical slices (0.1µm each). Scale bar 5μm.

**Figure S3. Cup is necessary for the formation of** ***cycA*** **and** ***cycB*** **mRNPs.** 

**(A)** Co-visualization of *cycA* and *cycB* mRNAs via smFISH in stage 6 egg chambers expressing Bru1-GFP in indicated backgrounds. White box indicates the location of the zoomed in images. Images are deconvolved, XY max-intensity Z-projections of 5 optical slices (0.3µm each). Scale bars, 10μm.

**(B)** Overlap volume ratio analysis of *cycA* and *cycB* mRNAs with Bru1-GFP performed in *control* and *cup1*^*RNAi*^ stage 5-7 egg chambers (****p<0.0001; *control* vs *cup1*^*RNAi*^ : n=11 and n=27).

**Figure S4.  Bru1 overexpression rescues mitotic re-entry of** ***cup*** **mutant egg chambers.**

**(A)** Several ovariole chains of the indicated backgrounds with ɑ-tubulin visualized via IF. Images are deconvolved, XY max-intensity Z-projections of 5 (*UAS-bru1-GFP*), 6 (*cup*^*1/01355*^) and 3 (*cup*^*1/01355*^*; UAS-bru1-GFP*) optical slices (0.3µm each). Scale bars, 100μm.

 **(B)** Chain of *wildtype* egg chambers expressing Bru1-GFP with CycB visualized via IF. Membrane (WGA). Images are deconvolved, XY max-intensity Z-projections of 5 optical slices. Scale bar 50μm.

**Figure S5.** ***cycA*** **and** ***cycB*** **mRNAs accumulate in Me31B-bodies in a Bru1 and Cup-dependent manner.** 

**(A)** Co-visualization of *cycA* and *cycB* mRNAs via STED in the nurse cells of a sectioned egg chamber at mid-oogenesis, expressing Cup-YFP and Me31B-GFP.  White box indicates the location of the zoomed in images in Figure 5A. Images are deconvolved, XY max-intensity Z-projections of 3 optical slices (0.1µm each). Scale bar 1μm.

**(B)** Co-visualization of *cycA* and *cycB* mRNAs and Bru1 via smFISH and IF in the nurse cells of a sectioned egg chamber at mid-oogenesis, expressing Cup-YFP and Me31B-GFP. White box indicates the location of the zoomed in images in Figure 5B. Images are deconvolved, XY max-intensity Z-projections of 5 optical slices (0.2µm each). Scale bar 1μm.

**(C)** Co-visualization of *cycA* and *cycB* mRNAs in stage 5 egg chambers expressing Me31B-GFP in indicated RNAi backgrounds. White box indicates the location of the zoomed in images in Figure 5C. Images are deconvolved, XY max-intensity Z-projections of 4 (*control*), 5 (*cup*^*RNAi*^) and 5 (*bru1*^*RNAi*^) optical slices (0.3µm each). Scale bars, 10μm.

**Figure S6. Me31B differentially regulates the translation of** ***cycA*** **and** ***cycB*** **mRNAs, but their association with Cup-YFP is independent of Me31B.**

**(A)** Co-visualization of *cycA* and *cycB* mRNAs via smFISH and F-actin in sectioned mid-oogenesis egg chamber expressing Cup-YFP and Me31B-GFP. F-actin was stained to visualize the membrane and the ring canals. *cycA* mRNA, *cycB* mRNA, and Cup accumulation at the ring canals in *me31B*^*RNAi*^ (red arrows). Images are deconvolved, single optical slices. Scale bars, 10μm.

**(B)** Co-visualization of *cycA* and *cycB* mRNAs and Bru1 protein via IF in sectioned mid-oogenesis egg chambers expressing Cup-YFP and Me31B-GFP in *control* and *me31B*^*RNAi*^ backgrounds. *cycA* mRNA, *cycB* mRNA, Bru1 and Cup accumulation (red arrows). 3D Surface plots depict the signal intensities (0-255) using “5_ramps LUT”.  Images are deconvolved, XY max-intensity Z-projections of 5 (*control*) and 9 (*me31B*^*RNAi*^) optical slices (0.2µm each). Scale bars, 10μm.
