# Supplementary material for: Post-transcriptional regulation of cyclin A and cyclin B mRNAs is mediated by Bruno 1 and Cup, and further fine-tuned within P-bodies
Source: bioRxiv. 2024 Oct 17:2024.10.17.618951. Preprint. [Version 1] doi: 10.1101/2024.10.17.618951 (PMC11507948; doi:10.1101/2024.10.17.618951)
Supplement: Supplementary file 1 [file media-1.pdf]

Table S1. Probe sequences used in smFISH experiments.

Probes	*cycB* mRNA	*cycA* mRNA
1	gattctgcaaatcgcccaag	gaatcttaatgcccggattc
2	ctgcgatgggacgacttatg	ctgcttggtgttcttcactc
3	ttgagatccttggagtcttt	attgagcacggcgaaattgg
4	cgaacgcaaaaacgccgaca	gacgcggcacattgttgttg
5	tcttggtcggaacgcgattg	gtcacggaagacaactttcc
6	actttagtgggttctacagt	actccacattctcatcaaca
7	ctcggaagaactggacttga	caccacattcgatttcttgg
8	tttaaggtgggctcgttcac	aaggtcttgaattgctccac
9	agcgacttcttcgacagatt	tatcgttgttgtcctcatac
10	caaagcggcacgcagtttgg	tctctttgtcgactagggag
11	aaactcccatcacgggtttg	agcaccgaatttcacgtcat
12	tactggttcccgtcgaattc	agatcgtagtctaccagctc
13	tctgcctctttgcgggaaac	cacggacatgggtgttgaat
14	ttcttggtttctggcagttc	gacatgggggactgaacatc
15	ctttttcacttccagtgagt	atgacaccgagaatggagcg
16	taataaggggcatcctggtc	cacgctgatatcactggact
17	cgtagtgactgcactgttgc	cggaagctccttgactcttc
18	tggtgggcatcgtagatgtg	tactgaaccacttccaggaa
19	cgcttgctggaaagggacat	atattccagaatgtccatct
20	tcaatgtcctcgattccagc	cgatgtttcttctcgctctc
21	caggttctccttgtcattgg	ttctgtctgcgcatatagag
22	cgtttacatattcggagacc	agcgcatattgtggctaatg
23	tccacctgatacaagtagtc	accagccaatcaataaggat
24	caggtgatccttgtgaatgg	cagtttgtactcctcggaaa
25	caatcgatcagcacggctcg	aagaccgagagatagagcgt
26	actgcaggtggacttcgttg	ccgccatttggctcaaaaat
27	aaggtctctgcagccagatg	agctgtaacttggagcgcac
28	gtagcgatcaatgatagcca	caatatacatagctgccgtg
29	attgcaagtacgtgcgtttg	gggatagatttcctcgtatt
30	gctatgaagagtgctgtcac	gaagacaaactcaccgacct
31	ggaacagctcctcgtacttg	cttggtgtaactgtcgtcgg
32	gacgaaatctccgattgccg	ttcaagataacctgctccat
33	tgtaggtgtcgtccgtgatg	tgcacagatcgaaggagaga
34	ttgaagatttgcagctccat	atgaagacgtaagcagtcgg
35	cgcgacagattacagtcgat	aggcatgtcgcaaagtacag
36	aaggaagtgaatcggcagcg	tacagcgtcatgtacttcag
37	gtacttggacatcgtatggt	ccatgagcgacaactcggaa
38	ccacggaagctaactcgatg	tattgcaagtacgtttctcc
39	ctgtaagtggccatttcgta	cgactgaagcggatgacata
40	cgacaggaacagtgaggcag	acatctccatgcccaaaatg
41	tggtttccattgagcaagtg	agcttgtaagtcgtgatctc
42	acggtcgttgaatcctgtac	gcaacacaaccgtttttagg
43	gagtatcgcgagtagaaggt	gttttgtgggtgtgacacag
44	cgggtaatcggacgcaagtg	tattgtacttttcccgcatg
45	tggtacttgttgtagatggc	catagccaccttcttgtaag
46	cgatcttctggaacttgctg	ttgctcatctcaacggattc
47	cacaatcgagtccatcagcg	cacagagctgatcgaagtcg
48		tctttctgcttgcaattgta
