# Supplementary material for: Post-transcriptional regulation of cyclin A and cyclin B mRNAs is mediated by Bruno 1 and Cup, and further fine-tuned within P-bodies
Source: bioRxiv. 2024 Oct 17:2024.10.17.618951. Preprint. [Version 1] doi: 10.1101/2024.10.17.618951 (PMC11507948; doi:10.1101/2024.10.17.618951)
Supplement: Supplementary file 2 [file media-2.pdf]

Table 2. Primer sequences used in RT-qPCR experiments

CycA FWD	'GTCACACCCACAAAACGGCA'
CycA REV	'GCTGCTGGTGCTCATCCTCT'
CycB FWD	'CGAGGACGAGCACCATACGA'
CycB REV	'AGTGCAGCGACAGGAACAGT'
