# Supplementary material for: Post-transcriptional regulation of cyclin A and cyclin B mRNAs is mediated by Bruno 1 and Cup, and further fine-tuned within P-bodies
Source: bioRxiv. 2024 Oct 17:2024.10.17.618951. Preprint. [Version 1] doi: 10.1101/2024.10.17.618951 (PMC11507948; doi:10.1101/2024.10.17.618951)
Supplement: Supplementary file 4 [file media-5.pdf]

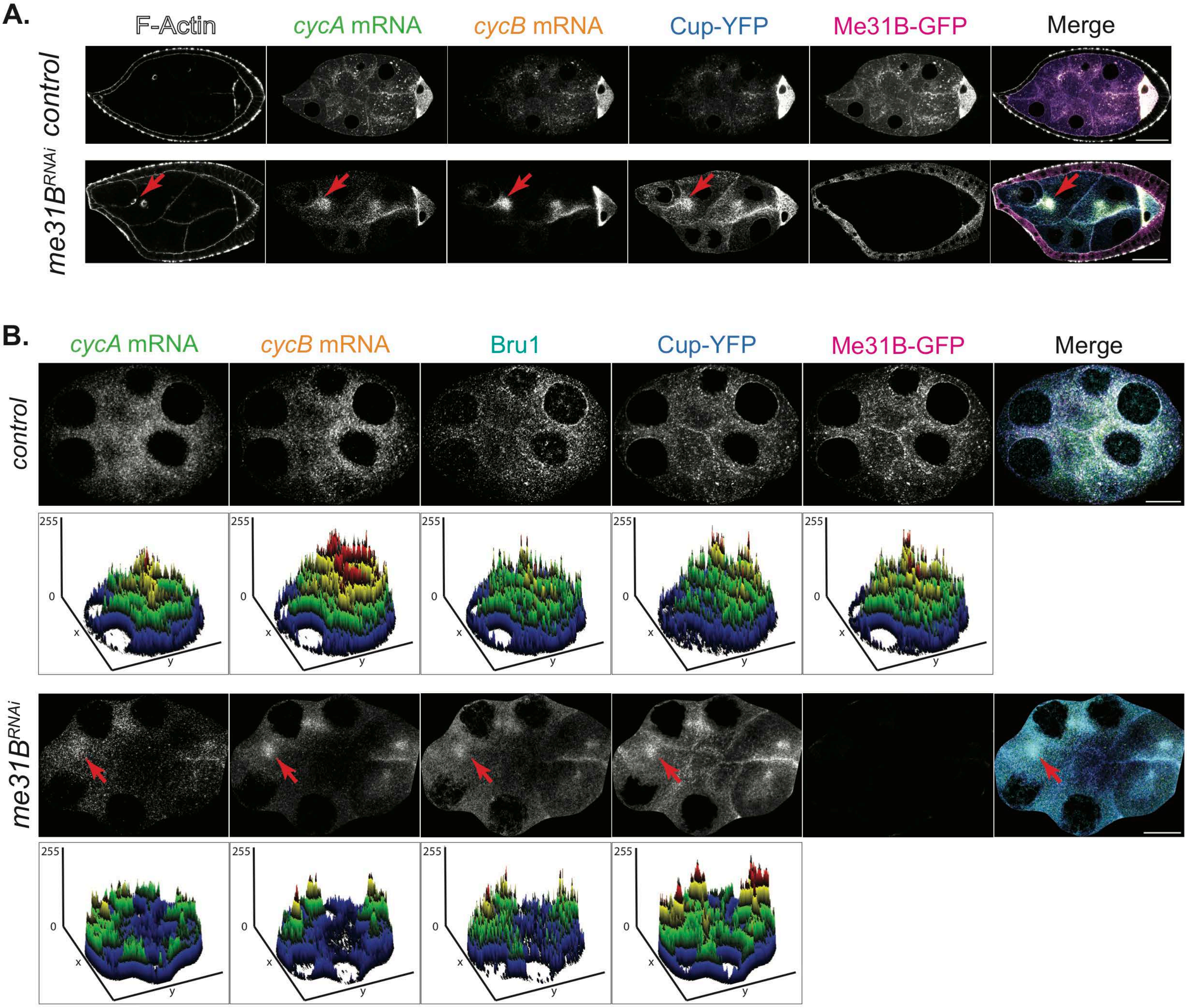
A.
F-Actin
cycA mRNA
cycB mRNA
Cup-YFP
Me31B-GFP
Merge
control
me31B^RNAi
B.
cycA mRNA
cycB mRNA
Bru1
Cup-YFP
Me31B-GFP
Merge
control
me31B^RNAi
255
0
x
y
