# Supplementary material for: Post-transcriptional regulation of cyclin A and cyclin B mRNAs is mediated by Bruno 1 and Cup, and further fine-tuned within P-bodies
Source: bioRxiv. 2024 Oct 17:2024.10.17.618951. Preprint. [Version 1] doi: 10.1101/2024.10.17.618951 (PMC11507948; doi:10.1101/2024.10.17.618951)
Supplement: Supplementary file 5 [file media-6.pdf]

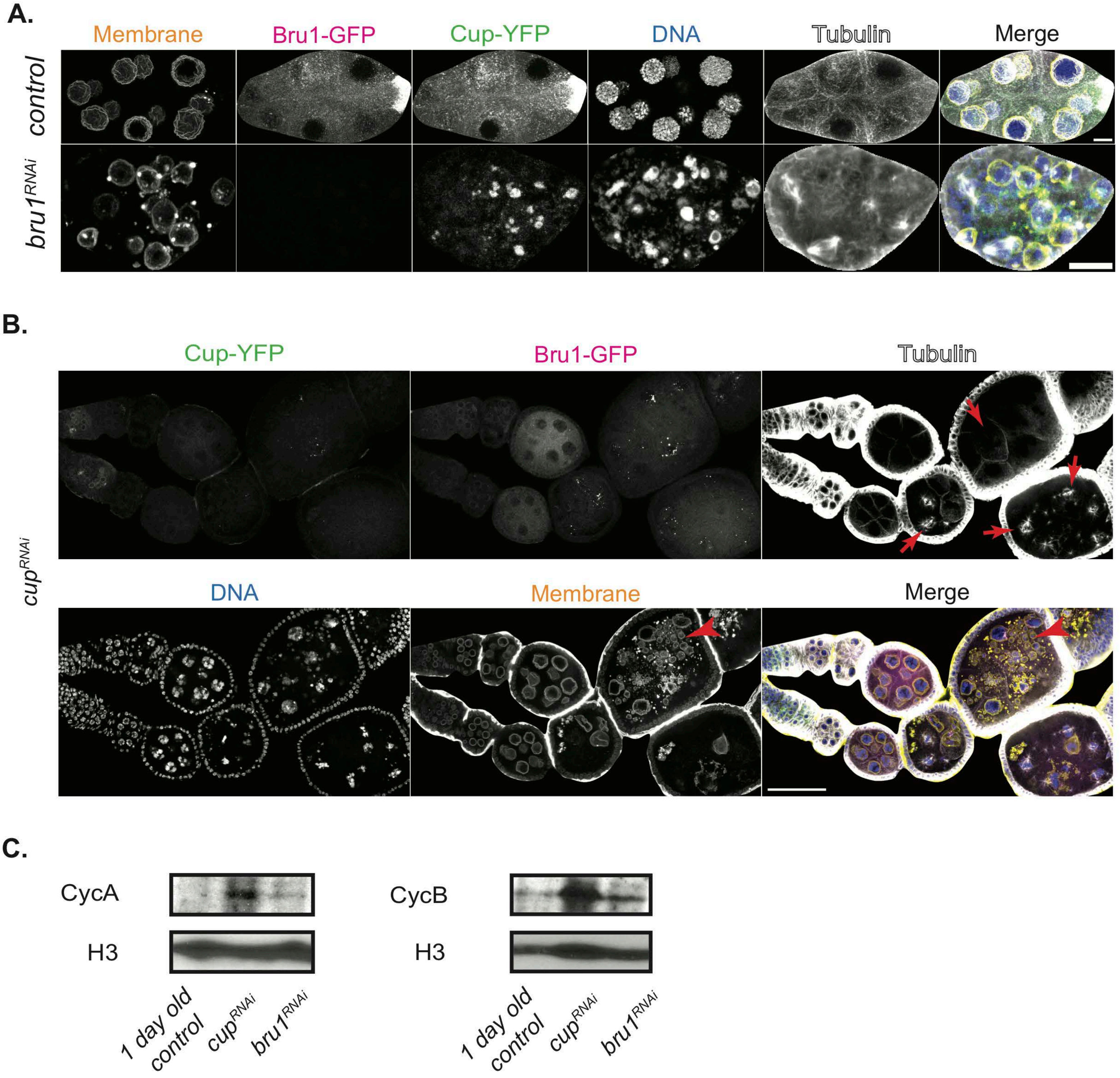

A.
Membrane
Bru1-GFP
Cup-YFP
DNA
Tubulin
Merge
control
bru1RNAi
B.
Cup-YFP
Bru1-GFP
Tubulin
cupRNAi
DNA
Membrane
Merge
C.
CycA
H3
1 day old control
cupRNAi
bru1RNAi
CycB
H3
1 day old control
cupRNAi
bru1RNAi
