# Supplementary material for: Post-transcriptional regulation of cyclin A and cyclin B mRNAs is mediated by Bruno 1 and Cup, and further fine-tuned within P-bodies
Source: bioRxiv. 2024 Oct 17:2024.10.17.618951. Preprint. [Version 1] doi: 10.1101/2024.10.17.618951 (PMC11507948; doi:10.1101/2024.10.17.618951)
Supplement: Supplementary file 6 [file media-7.pdf]

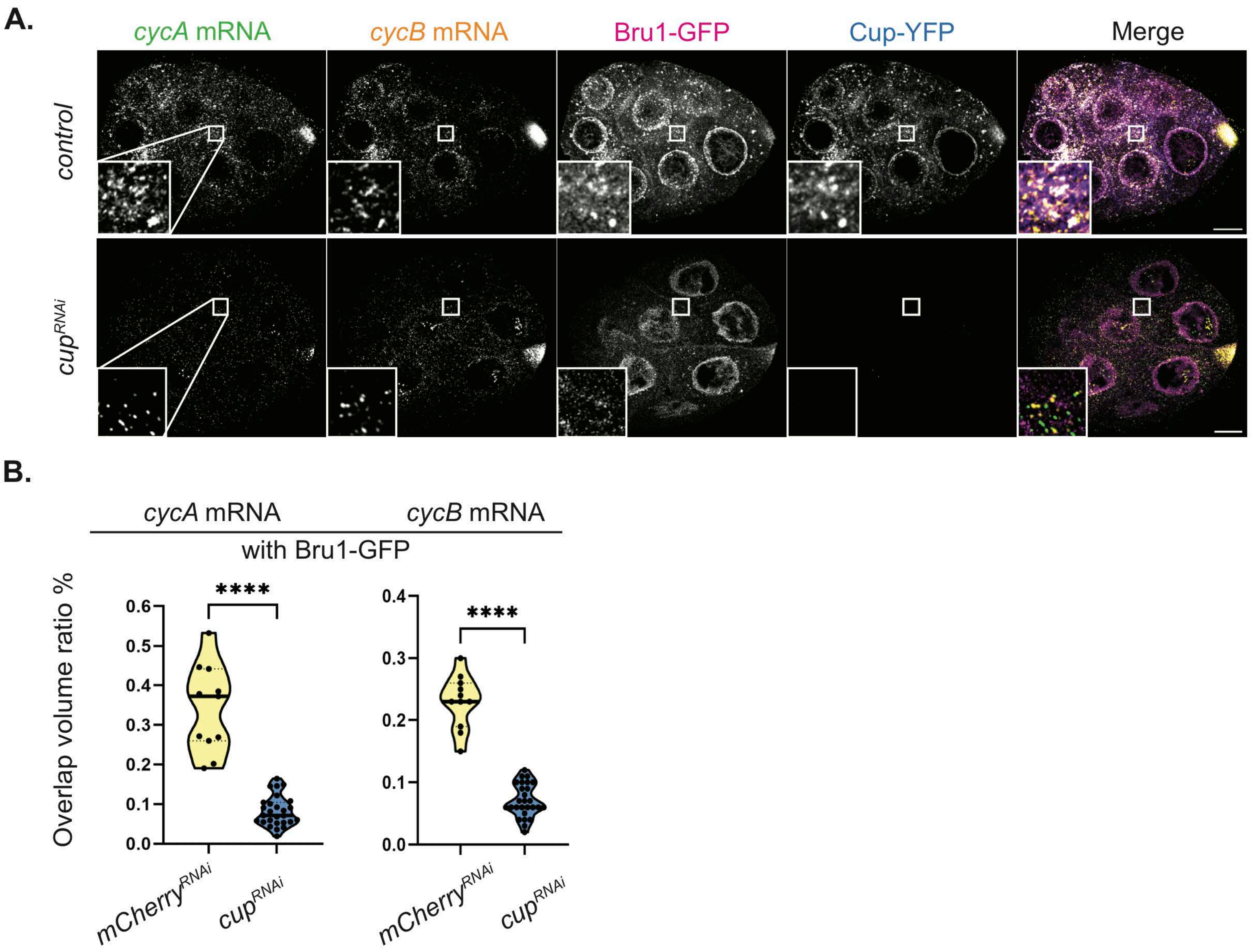

A.
cycA mRNA
cycB mRNA
Bru1-GFP
Cup-YFP
Merge
control
cupRNAi
B.
cycA mRNA
cycB mRNA
with Bru1-GFP
Overlap volume ratio %
****
****
0.0
0.1
0.2
0.3
0.4
0.5
0.6
mCherryRNAi
cupRNAi
