# Supplementary material for: Post-transcriptional regulation of cyclin A and cyclin B mRNAs is mediated by Bruno 1 and Cup, and further fine-tuned within P-bodies
Source: bioRxiv. 2024 Oct 17:2024.10.17.618951. Preprint. [Version 1] doi: 10.1101/2024.10.17.618951 (PMC11507948; doi:10.1101/2024.10.17.618951)
Supplement: Supplementary file 7 [file media-8.pdf]

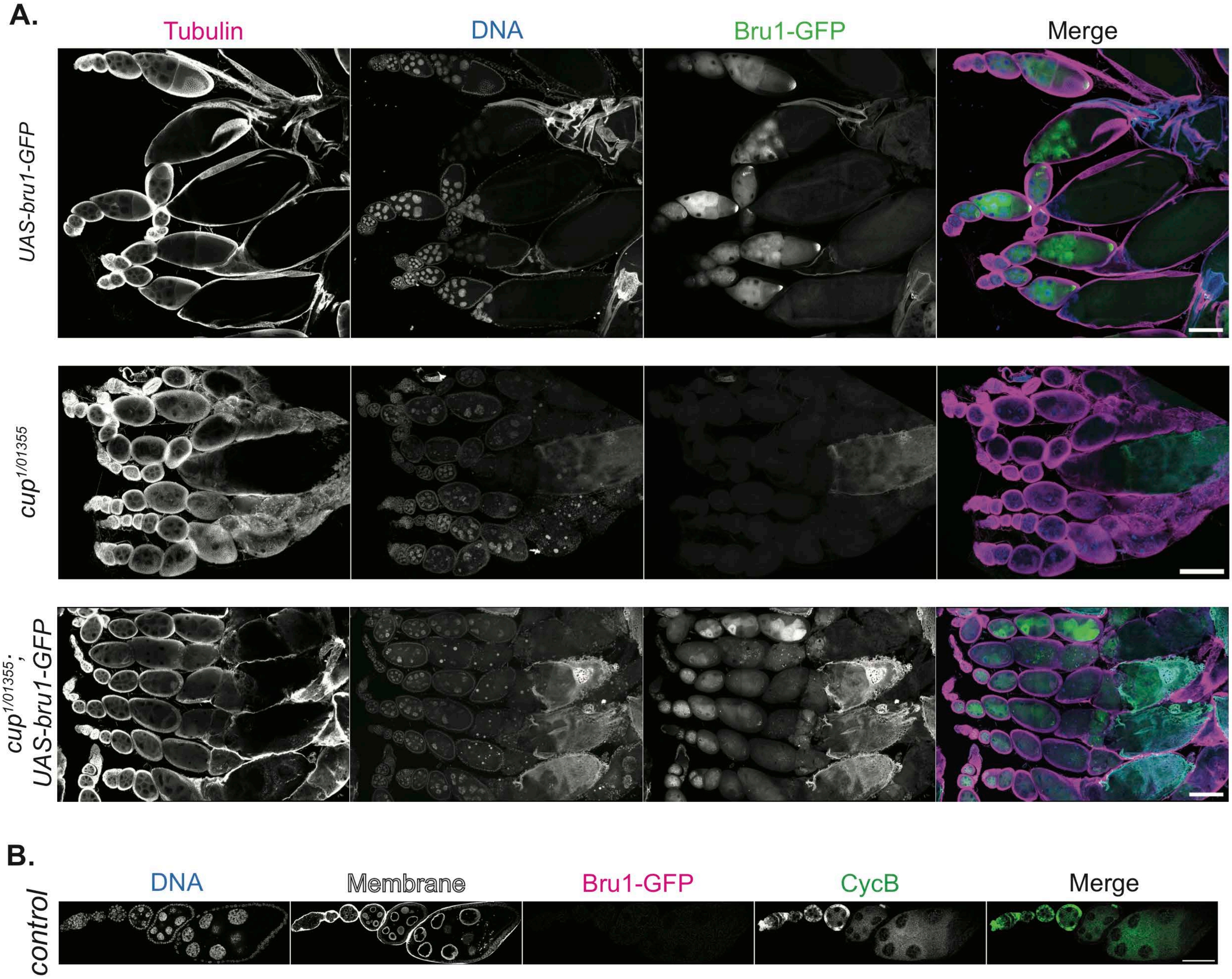
A.
Tubulin
DNA
Bru1-GFP
Merge
UAS-bru1-GFP
cup1/01355
cup1/01355;
UAS-bru1-GFP
B.
DNA
Membrane
Bru1-GFP
CycB
Merge
control
